# Having Reliable Support: A Prerequisite to Promote Sexual and Reproductive Health in Young Women with ADHD

**DOI:** 10.1007/s10508-024-03001-5

**Published:** 2024-09-23

**Authors:** Karin Wallin, Inger Wallin-Lundell, Siw Alehagen, Lena Hanberger, Sally Hultsjö

**Affiliations:** 1 Department of Obstetrics and Gynecology in Linköping, Department of Health, Medicine and Caring Sciences, Linköping University, SE 581 83 Linköping, Sweden; 2 Department of Health, Medicine and Caring Sciences, Linköping University, Linköping, Sweden; 3 Department of Health Sciences, Swedish Red Cross University, Huddinge, Sweden; 4 Department of Psychiatry, Ryhov County Hospital, Jönköping, Sweden

**Keywords:** ADHD, DSM-5, Phenomenography, Sexual and reproductive health, Young women

## Abstract

Living with attention deficit hyperactivity disorder (ADHD) may influence sexual behaviors and intimate relationships in young women, resulting in a higher risk of unwanted pregnancy, sexual victimization, and sexual dysfunction. To develop adequate support, the study aimed to describe conceptions of how to promote sexual and reproductive health (SRH) in young women with ADHD. A secondary analysis using phenomenography was performed on qualitative interview data exploring variations of support. The study involved 15 young women with ADHD, aged 15–29 years, and 16 health care professionals, with various professions, working in the fields of gynecology, youth health, and psychiatry. Having reliable support was conceptualized as fundamental for promoting SRH. Access to information concerning SRH and living with ADHD as well as early support from health care contributed to a reliable support enabling self-knowledge and management of sexual relationships. Trustful relationships in health care were perceived as important because of previous experiences of feeling misunderstood and criticized in life, making them feel comfortable discussing SRH. Clinical encounters with a clear structure were further perceived to make information more accessible and clinics that provided appropriate organizational conditions and collaborated with other clinics were described to enhance the availability of support. This study reveals the need for clinics to provide conditions that ensure SRH support is available, accessible, and free of stigmatization. Early intervention programs for young women with ADHD may be considered, offering guidance on SRH issues in both psychiatric and sexual health clinics.

## Introduction

Women with attention deficit hyperactivity disorder (ADHD) may be at risk of compromised sexual and reproductive health (SRH) in young adulthood, which demonstrates a need for individualized support that can promote healthy sexual behaviors and relationships and prevent sexual risk-taking behaviors and sexual victimization (Young et al., 2020). ADHD is a common neurodevelopmental disorder affecting approximately 5–7% of children and adolescents and 3% of women (Fayyad et al., 2017; Willcutt, 2012). Symptoms of inattention and/or hyperactivity and impulsivity are associated with impairment in daily life function during the lifespan, affecting academic and occupational performance, relationships, and risk-taking behaviors negatively (American Psychiatric Association, 2013; Merrill et al., 2019). Differences in how symptoms are presented based on gender, and along with the presence of common comorbid conditions such as autism spectrum disorder (ASD), personality disorders, and depressive and anxiety disorders, could mask ADHD symptoms in young women and delay assessment and diagnosis (Fuller-Thomson et al., 2016; Young et al., 2020). In Sweden, women receive an ADHD diagnosis and treatment approximately four years later than men, at the age of 23.5 years (Skoglund et al., 2024).

Even though casual sexual relationships have been described as leading to positive sexual experiences for young women with ADHD (Wallin et al., 2022), the challenges they face in forming intimate relationships, sexual dysfunction, and taking sexual risks may also negatively affect their SRH compared to young adults without a diagnosis (Young et al., 2020). Living with ADHD has been related to early sexual initiation, and having unprotected sex or sex with multiple partners in young women with ADHD (Berry et al., 2021; Hechtman et al., 2016; Hosain et al., 2012), resulting in higher risks of unplanned pregnancy at a young age, receiving a sexually transmitted disease (STD), and being sexually victimized (Chen et al., 2018; Huggins et al., 2015; Skoglund et al., 2019; Snyder, 2015). However, Marsh et al. (2015) discovered that even if young adults with ADHD were not anxious about engaging in sexual activities, they may experience fear of intimacy and lower expectations of close relationships. Young women also reported having shorter romantic relationships and lower relational quality than women without a diagnosis (Bruner et al., 2015; Rokeach & Wiener, 2018). Furthermore, studies exploring sexual function revealed that young women with ADHD experienced more difficulties in reaching orgasm, less sexual satisfaction, and more pain, compared to adults without a diagnosis (Bijlenga et al., 2018; Jabalkandi et al., 2020).

Despite evidence of adverse negative outcomes on SRH, only a few studies emphasize how to promote healthy sexual behaviors and intimate relationships in young women with ADHD (Klint Carlander et al., 2022; Young et al., 2020). A Swedish study described the importance of targeting contraceptive counseling in youth clinics specialized in SRH, suggesting that there is a need for development of guidelines and enhancing midwives’ knowledge to better address the women’s needs (Klint Carlander et al., 2022). Young et al. (2020) further proposed additional sexual health education for young women with ADHD and ADHD training in health care providers in sexual health clinics. Good educational performance and having a qualitative relationship with one’s mother have also been reported to reduce unplanned pregnancies and numbers of sexual partners, emphasizing the need for early support in schools and families (Huggins et al., 2015; Owens & Hinshaw, 2020).

To develop sufficient support and individualized health care encounters for young women with ADHD, it is necessary to have a better understanding of how healthy sexual behaviors and relationships can be. Understanding how SRH can be promoted may therefore contribute to the development of targeted interventions and improve health outcomes for this population. The current study may also endorse person-centered care, which health care systems worldwide strive to integrate to improve well-being, satisfaction with care, and efficiency in health care (Duong et al., 2024). Person-centered care emphasizes treating patients as equals, actively involving them in decisions about their own health (Ekman et al., 2011). Enhancing understanding of the women’s challenges and needs may help HCPs improve communication with women, provide a non-judgmental environment, and tailor support according to their unique preferences, needs, and values. Improving involvement in care may further strengthen self-belief and personal resources (Ekman et al., 2011), which, according to Pender’s health promotion model, are factors that influence choices of healthy behaviors (Murdaugh et al., 2019).

In Sweden, youth clinics and gynecology clinics in public health care provide counseling concerning SRH and testing for STDs, free of charge in. The clinics are mostly run by midwives, gynecologists, and counselors or psychologists (Association of Youth Clinics in Sweden, 2018; Public Health Agency of Sweden, 2020). Assessment of ADHD as well as medical treatment, counseling, and psychoeducational interventions are provided mainly by psychiatric nurses, occupational therapists, psychologists, and psychiatrists in outpatient psychiatric clinics belonging to public health care clinics (Swedish Association of Local Authorities & Regions, 2023).

To reach a comprehensive description that capture variations in how SRH can be promoted, this study included both young women with ADHD and HCPs with experience of counseling these women in areas of SRH. While women with ADHD have a lived experience, HCPs working in psychiatric clinics, youth health, and gynecological clinics meet young women with ADHD on a daily basis. Insights from both groups can lead to the development of interventions that are aligned with the women’s needs and the professionals’ practical everyday work.

Thus, the aim of this study was to describe conceptions of how to promote SRH in young women with ADHD.

## Method

This study included a secondary analysis of two previous studies exploring experiences and perceptions of SRH in young women with ADHD (Wallin et al., 2022, 2024). Secondary analysis of qualitative data provides an opportunity to capture new perspectives on previously collected data. It can include examining existing data from a different theoretical perspective, challenging or validating earlier findings, or applying a new research question (Tate & Happ, 2018). In addition to descriptions of experiences and perceptions, the data from the two previous studies also included statements about different types of support and its importance for SRH. Therefore, a secondary analysis was found appropriate for this study as the purpose was to search for new perspectives by answering a new research question focusing on how to promote SRH.

### Participants

In the present study, the same participants as in our two primary studies were included (Table 1). The studies used purposive sampling; the first included women with ADHD aged 15–29 years (Wallin et al., 2022), and the second included HCPs with experience of meeting young women with ADHD in their daily work (Wallin et al., 2024). The women and HCPs were recruited from youth clinics, gynecological clinics, and outpatient psychiatric clinics in one university hospital, three regional hospitals, and three primary care facilities all situated in the southeast of Sweden. The women were also recruited from Facebook pages belonging to interest organizations for ADHD. To ensure the women’s ADHD diagnoses, all participating women were asked when and from whom they received their diagnoses.

**Table 1:** Characteristics of participants

Variable	Number
Participants, n	31
Women with ADHD, n	15
*Type of ADHD*	
Combined	13
Predominately attentive	2
Age of women with ADHD, median age (min–max)	25 (15–29)
Health care professionals, n	16
*Profession*	
Midwives	7
Occupational therapist	1
Psychiatric nurses	4
Psychologists	2
Counselors	2
Years in health care profession, median year (min–max)	12.5 (2–40)

### Measures and Procedure

The two studies used qualitative interviews with an inductive approach. Both studies were carried out by the authors presenting the current manuscript. The studies, with women and HCPs, respectively, used interviews that were carried out individually and in focus groups. Both studies analyzed data using reflexive thematic analysis according to Braun and Clarke’s (2006, 2019) six-step guide. Semi-structured questions, including questions about the women’s self-experience or the HCPs’ perceptions and experiences of SRH, guided the interviews (Table 2). For example, the guides covered domains concerning intimate relationships, sexuality, and how ADHD may influence SRH. Some questions also covered how to support SRH but where not included in the analysis of the primary studies. The questions were not covered in a specific order but followed the flow of the conversation. To enable the participating women and HCPs to express their perspective, the researchers tried to have an open mind, not valuing answers as right or wrong (Sjöström & Dahlgren, 2002). Instead, prompting questions like “Can you tell me more?” or “Can you explain?” were asked to clarify questions and reach a deeper understanding of the participants’ experiences. Interviews lasted for 24–80 min (median 45 min). They were all recorded and transcribed verbatim. The interviews with the women were performed between December 2019 and January 2021, and those with the HCPs between December 2019 and January 2022. The data collection was delayed several times due to COVID-19.

**Table 2:** Semi-structured questions in the two primary studies

Primary study I: Interviews with young women with ADHD	
Starting question	Can you tell me what sexual health means to you?
Sexuality	Can you tell me something about your sexuality that you appreciate?
Relationships	How do you view your sexual relationships? What are your challenges? Strengths? What is important for you to make causal/romantic relationships work?
Sexual situations	Can you tell me about a sexual situation with yourself or with someone else that felt good/did not feel right? What contributed? What could have helped you to make it better?
ADHD	Do you think ADHD can affect your sexual and reproductive health? In what ways?
Needs	What do you need to experience sexual and reproductive health? Who do you turn to for support?

Primary study II: Interviews with health care professionals
Main questions
Can you tell me about your experiences of talking to young women with ADHD about sexual and reproductive health?
Can you describe subjects concerning sexual and reproductive health that are raised by the women? Which questions do you perceive to be important to raise?
In your experience, what role does ADHD play on sexual and reproductive health for the women?
Can you tell me about a conversation concerning sexual and reproductive health with a young woman with ADHD that you are pleased with? What worked out well?
Can you tell me about a conversation concerning sexual and reproductive health with a young woman with ADHD that you found challenging?
What kind of support or counseling do you perceive to help women with ADHD to promote their sexual and reproductive health?
Probes
Can you tell me more? Can you explain?

### Secondary Data Analysis

In this secondary analysis, a phenomenographic approach was chosen as it aims to understand a phenomenon by gaining knowledge of how it is experienced and conceived. In phenomenographic research, it is assumed that individuals differ in how they perceive their world. These differences and similarities can be described and understood by others generated through analysis of semi-structured interviews (Marton et al., 1988). Emphasizing variation in research could be one way of raising awareness of individuals’ diverse needs to support health (Sjöström & Dahlgren, 2002) and may therefore be a useful approach to answer the aim of this study.

All data from both primary studies were included, to provide a variation of description. The data analysis followed the structure of phenomenographic analysis described by Sjöström and Dahlgren (2002). The authors began by reading all the manuscripts from the primary studies to become familiar with the data. In the next step, significant elements that answered the research question, describing conceptions of how to promote SRH in young women with ADHD, were highlighted in all transcripts. Longer statements were condensed to illustrate the central parts. Thereafter, statements were classified together in preliminary categories, followed by a preliminary comparison of categories, where the researchers tried to establish borders between them. The preliminary comparison entailed the researchers to revise some of the categories and validate them by going back to codes and original data. The last two steps included naming the categories to emphasize their essence and a contrastive comparison of categories, in which the unique characters of each category were described as well as the resemblances between categories, revealing the outcome space of analysis.

To enhance credibility in phenomenographic research, the researcher needs to show that categories are well supported by the data (Sjöström & Dahlgren, 2002). Credibility was strengthened by providing a precise description of the research process. The authors also had experience of performing and analyzing qualitative data, and all were involved in the methodological process. K.W., I.W.L., and S.H. coded and classified the preliminary categories. However, all interview data were read by all authors and categories were discussed and agreed upon together in the group, which improved the fit between the data and results. Representative quotes from the data further illustrated and supported the results.

## Results

**Fig. 1:**
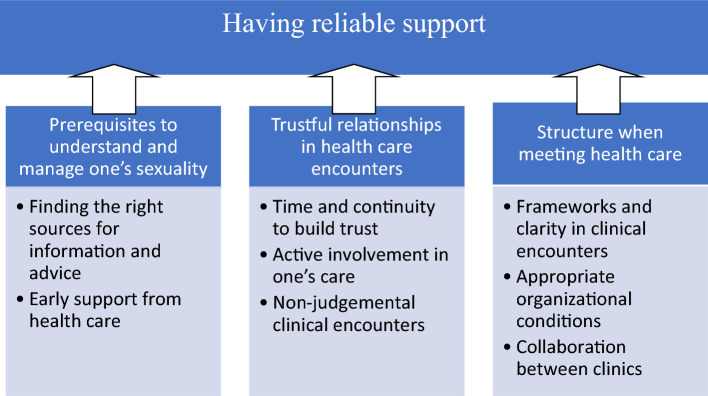
Outcome space—categories and how they relate to each other

In phenomenography, the result consists of the outcome space, which is a description of categories and their relation to each other (Sjöström & Dahlgren, 2002). In this study, the outcome space consisted of one overreaching category and three categories (Fig. 1). The three categories were distinct from each other but highlighted different aspects that together were needed to achieve the overreaching category of having reliable support. Therefore, the outcome space could contribute to insights on how to promote SRH in young women with ADHD.

### Having Reliable Support

This overreaching category illustrated that having reliable support was considered important to promote SRH in young women with ADHD. Having reliable support was described in the first category as having the right conditions to manage one’s sexuality. While knowledge and support concerning SRH could be found on Internet, among friends or improved by early recognition of ADHD symptoms, the women were also dependent on their interactions with healthcare to receive sufficient support. The other two categories described different aspects of reliable support in health care: the importance of a trustful relationship in health encounters and the need for a functional structure in meetings and organization.

### Prerequisites to Understand and Manage One’s Sexuality

Most of the women and HCPs perceived that it was important for the women to have the right support and guidance to rely on to enable understanding of their own sexuality and management of sexual relationships in relation to having ADHD. Early recognition of symptoms of ADHD and obtaining a diagnosis were described by both women and HCPs as prerequisites for early support from health care that could prevent sexual risk behaviors and strengthen women’s self-esteem.

### Finding the Right Sources for Information and Advice

Both the women and the HCPs perceived that it was important for women with ADHD to find the right sources of information and advice that suited them. Several of the women described that the primary source of information and advice on sexual relationships came from the Internet, friends, partners, or family. Friends could often help with advice, but they were described as sometimes lacking experience or understanding of how sexual relationships could be affected by living with ADHD. Therefore, it was perceived easier to receive support from an older sister with more experience of relationships than a same-aged friend or from a friend or mother with the same diagnosis. Several women and HCPs further described the information on the Internet as easily accessible, and some women believed it could provide answers about bodily functions and how sex worked. Pornography was mentioned by both women and HCPs as a common source of information but was also described by some women as unreliable because it did not give a true picture of sexual relationships and female desire. However, even though there were other sources on the Internet that were perceived as more reliable, these women described that it could be difficult to find suitable information that was based on experiences of young adults with ADHD. One woman said:

The thing about sex and having these diagnoses (ADHD+ASD) is that you have another thing that you can’t really ask anyone about. You somehow operate on a different set of rules than many others. You think you don’t, but it becomes difficult to relate to others when they talk because you don’t have the same experience with things. And when you read articles about how it’s supposed to be, you don’t recognize yourself. In my opinion, there is no information available to make my life a little better. (Informant 9)

To find appropriate information, both the women and the HCPs described that information needed to be easily available and accessible in many different forms. Some HCPs at the youth clinic perceived that it was especially important to make information about SRH available in many different places so it could be accessed by women with ADHD when they were interested and motivated. They thought that written information should be available in waiting rooms and schools, but also in other forms such as podcasts, apps, and websites administered by HCPs. Several of the women described that if information and advice from friends, family, or the Internet was not sufficient, they would rely on contact with health care.

### Early Support from Health Care

Both the women and the HCPs described the need for schools, parents, and health clinics to acknowledge symptoms of ADHD early in life to enable diagnosis, treatment, and support. According to most of the women and the HCPs, early contacts with psychiatry and youth clinics were perceived to contribute to women receiving help with ADHD medication and strategies to manage symptoms, which in turn could lead to less sexual risk-taking. They also perceived early support as a prerequisite for preventing low self-esteem and strengthening self-belief. Low self-esteem and fear of being rejected were described as making the women hesitate to express wishes and needs in both sexual situations and romantic relationships. A psychologist working in a psychiatric clinic discussed the importance of early support:

I think she would have needed quite a lot of support early on, preventively. Someone should have talked about relationships and sexual health when she was quite young. She should have had that with her in everyday life. It’s like life skills in a way. (…) What could affect the girls in particular is that they receive a diagnosis later. You have a few lost years where you could have worked more preventively. (Informant 16)

To support women early in life, several of the HCPs thought it was important to offer partners the opportunity to participate in counseling and for parents to have access to education on SRH and ADHD. Furthermore, some of the women and HCPs described benefits of meeting other women of the same age with the same diagnosis. Like this woman expressed, other women could help to improve one’s understanding of their sexuality and help with advice:

When I talk to people who have similar diagnoses, you can somehow understand why it hurt, because I have been in this situation, and it could be about sex or about work. “Yes, but I understand you, do it like this next time!” That you become each other’s psychologists to solve everyday problems. (Informant 9)

HCPs from psychiatric clinics thought that expanding existing psychoeducational groups for adults with discussions about sexuality could be a way to promote SRH. They also perceived that there might be a need for discussion groups on sexuality in adolescence, and that homogeneous groups consisting only of girls with the same diagnosis could facilitate understanding and a conducive discussion environment.

### Trusting Relationships in Health Care Encounters

Several of the women found it important to be able to discuss sexuality and relationships with HCPs. Trusting relationships in health care were described by both the women and the HCPs to be essential for women to feel comfortable when discussing SRH and their ADHD diagnosis. It was perceived to be particularly important that women with ADHD could rely on being listened to and taken seriously, as several had experiences of feeling misunderstood and criticized by adults in both school and health care.

### Time and Continuity to Build Trust

According to most of the women and the HCPs, it was important to have time to build a relationship in health care encounters, as it could help women develop a sense of trust in HCPs. In youth clinics and gynecological clinics, several of the HCPs described that a trusting relationship allowed women to feel comfortable discussing their sexual experiences and made them more likely to return for STD or pregnancy testing. However, HCPs at these clinics considered that the relatively short meetings could make it harder to have time to establish trust. Both the women and the HCPs also described that young women with ADHD could have difficulty building trust because of experiences of feeling mistrusted themselves. Some of the HCPs therefore perceived it to be especially important for women with ADHD to be followed up on multiple occasions, preferably with the same HCP, to establish a relationship.

Some of the women described that although a trusting relationship could make them rely on the midwife or doctor at the youth clinic or gynecological clinic, it was less important when they only intended to obtain contraception or get tested there. Instead, the women perceived that longer meetings with a counselor at a youth clinic or a psychologist or nurse at a psychiatric clinic were often necessary to discuss negative sexual experiences or relationship problems. Longer clinical relationships allowed time to get to know each other and contributed to understanding of the woman and her life situation, which strengthened the relationship and built trust. The importance of building trust was expressed by one woman like:

I talk to my psychologist. I would never go and talk at the youth clinic. It needs to be with someone I have built a relationship with. I go to the youth clinic for the practical aspects, no talking or anything like that, that’s not what I want. (Informant 6)

Some women and HCPs also described that longer counseling periods were important to process underlying causes of unhealthy sexual relationships and receive help with useful strategies. However, most of the HCPs working in psychiatry argued that there was a need to be able to offer more women longer counseling periods than was currently being done.

### Active Involvement in One’s Care

Several of the women and the HCPs described that support from HCPs should be based on the women’s needs and should involve them in decisions regarding their own SRH. Involvement could contribute to meaningful support and create trust in HCPs. In turn, some of the women described that trust in HCPs made them feel confident in making their own decisions based on the HCPs’ knowledge. In health care encounters, all women and HCPs describing health encounters perceived that feeling listened to and taken seriously was important because young women with ADHD often had experiences of feeling misunderstood and criticized by adults in schools and health care. When it came to contraceptives, some of the women considered it to be important to be asked about previous experiences as well as wishes and fears. Several of the women described experiences of feeling depressed when taking hormonal contraceptives and others spoke of difficulties in remembering to take birth control pills. One woman exemplified how she wanted to be treated by HCPs to feel involved in contraceptive counseling:

To be listened to. What do I want, what do I need? How do you feel when you take birth control pills, what contraceptive methods have you tried, and then ask if you have any other relevant medical conditions. Had this been done, I could have eliminated most possibilities right away. (Informant 5)

Some HCPs described that it was significant for the women to be allowed to be experts on their own diagnoses and to have the opportunity to choose contraceptive methods, without having HCPs questioning their choice. To contribute to involvement and maintain trust, the HCPs explained that they had to accept the woman’s choice of contraceptive, even if they did not always agree with it.

Most of the HCPs at youth clinics and gynecological clinics perceived a need to be flexible and not always follow predetermined frameworks to contribute to participation. They described that women with ADHD particularly needed the opportunity to choose the type of contraceptive follow-up they desired in order for the meetings to take place (phone call, video call, or clinic visit), or to receive reminders via SMS to arrive on time. Offering drop-in appointments and attending to women with ADHD who turned up at youth clinics without a booked appointment were also considered to be important as these women were described as often missing appointments or being late. The importance of flexibility was described by a counselor working in a youth clinic:

I think we need to be better at assuming that people function differently, that we have flexibility from the very beginning regarding the support that we provide. That we try to see things that might work for most people, for example keeping track of time, doesn’t work for everyone. (Informant 13)

### Non-Judgmental Clinical Encounters

To not feel judged in clinical meetings was perceived by most of the women and the HCPs as essential to form trustful relationships that provided an open and reliable environment. Sexual behaviors in young women with ADHD were described as sometimes breaking sexual norms. It was therefore perceived especially important for HCPs to not criticize or patronize them when discussing their sexual behaviors. Some of the women mentioned the importance of being approached by HCPs like any other adult. Others expressed, in accordance with this woman, for HCPs to reassure the positive aspects of their sexual behavior:

I went to two psychologists. They didn’t judge me, they were positive. “Of course, you should have sex with who you want. There’s nothing wrong with that” which felt good. But the thing is, they told me that I should have sex when I'm feeling well. That's the point. So, it was important that they emphasized that. It was reassuring to hear. (Informant 6)

Some of the HCPs exemplified that by focusing on the fact that women showed responsibility in coming to test for STD or pregnancy, instead of focusing on negative outcomes of impulsive behavior, they could avoid feeling judged by HCPs. They also perceived that shame and self-criticism associated with feeling judged could be reduced by highlighting that difficulties with impulse control were not about bad morals. A midwife working in a youth clinic further elaborated on the importance of providing a non-judgmental relationship where the women could feel secure:

Many of these young women have experienced feelings of failure and criticism from adults. They have often been labeled as careless or irresponsible, which has caused them a great deal of distress. Therefore, it is crucial for you to be that adult figure who can make the woman feel safe and secure. Our entire organization is based on being those adults whom they can turn to and feel safe with when they are in need. That is why it is essential to build an alliance (Informant 14)

### Structure When Meeting Health Care

While most of the women and the HCPs perceived that women with ADHD could benefit from a clear structure in the clinical encounters, some HCPs also emphasized the advantages of structure at an organizational level. A comprehensible structure could enable women with ADHD to rely on the health care system and to receive adequate support and advice.

### Frameworks and Clarity in Clinical Encounters

Both the women and the HCPs described challenges for young women in concentrating during clinical encounters concerning SRH, related to symptoms of ADHD. Structure in health care encounters was therefore perceived to be especially important for them to stay focused during appointments and to absorb the provided information. Some HCPs thought that clarifying goals in the meeting and presenting time frames for what needed to be covered could help HCPs to redirect the conversation since the women sometimes tended to stray too far off-topic. Other HCPs described that visual aids could further clarify the planning of the conversation. They also saw a need to adapt appointment times to help the women to focus and assimilate information by offering longer appointments or several shorter meetings. Most of the women and the HCPs described that information provided, such as about contraception, had to be informative and concise to contribute to clarity. It was considered to be advantageous to provide information both orally and in writing. A midwife working with abortion and contraceptive counseling in a gynecological clinic expressed:

To be clear and structure the conversation. That you give both oral and written information. That they get a chance to ask again and that you can explain once more. That you allow it to take extra time. (Informant 2)

Both the women and the HCPs described that having ADHD also made the women more easily distracted. Reducing external stimuli in both the waiting room and the consultation room was therefore perceived to further facilitate focus during the appointment. According to HCPs, the waiting room could be filtered for unnecessary impressions. Some of the women described the importance of HCPs providing a calm and reassuring impression and not being disturbed by phones, while some of the HCPs emphasized the importance of not having too many people in the consultation room.

### Appropriate Organizational Conditions

Most of the HCPs and some of the women described that providing organizational conditions, like education and time, influenced the promotion of the women’s SRH. While some of the HCPs working in psychiatric clinics described a need for more education to feel confident in answering questions about SRH, others believed that schedules that did not permit sufficient time for appointments led to reluctance to raise questions concerning SRH. However, they perceived that introducing routines at the psychiatric clinic, asking all women with ADHD about SRH in the same way as, for example, violence or suicide, could promote better support. A psychiatric nurse working in a psychiatric clinic described the challenge of raising questions concerning SRH and the need for routines:

It’s uncomfortable. You don’t really know what to do with the answer. What am I supposed to say if they have severe problems with their sexual health? (…) We need clarity. We do have a template with questions to ask about life situation and if you have a partner. But there is no explicit question about sexual health. (…) If we had that as a part of our questionnaire, eventually we would not find it so difficult. But we need to do it routinely. (Informant 10)

In turn, some women perceived that general knowledge of ADHD and autism among HCPs in youth clinics and gynecological clinics was important to understand them better when talking about their sexual behavior. One of the women also suggested to routinely letting young adults fill out written forms in youth clinics and gynecological clinics regarding previous experiences of sexual victimization. According to her, this could be a way to destigmatize and facilitate discussions about more difficult topics.

### Collaboration Between Clinics

Most of the women and the HCPs described that being young and having ADHD involved having contact with various clinics within health care where different HCPs provided support with specific aspects of SRH, such as contraceptive counseling, abortion counseling, or discussions about self-esteem and sexual relationships. A psychologist working in a youth clinic expressed her perception of the various support needs and the benefit of collaboration between clinics:

They often need a combination of habilitation efforts. They need to learn how to function with their conditions. But there might also be mental illness, relationship problems and sexual abuse. There is a need for a unit that works with everything. (…) one and the same place where they don’t have to be shuffled around as they are currently. (Informant 4)

None of the women said that collaboration between clinics could promote their SRH; however, the HCPs found it of great importance. They perceived that a clear structure regarding which clinic was responsible for which aspect of treatment and support could improve the women’s care. Designating a clinic that had an overall responsibility with a comprehensive understanding of the woman’s life situation, as well as selected contact persons at other clinics, was perceived to create better support and to facilitate access to health care. According to the HCPs, clinics rarely collaborated, and they described a need to gather more health care authorities in the same place to facilitate collaboration.

## Discussion

The outcome space suggested that conceptions for promoting SRH in young women with ADHD can be assumed to resemble the support that most young adults need. However, reliable support was particularly prioritized by young women with a diagnosis. The participating women and HCPs shared a similar understanding of what was important to achieve reliable support, even though reflecting upon the support in various ways. To receive reliable support, there was a need to make information and advice concerning SHR more available and tailored to suit young women with ADHD. Receiving an ADHD diagnosis early in life was also perceived to enable treatment and support from psychiatric care and youth clinics, facilitating the prevention of sexual risk-taking and promotion of self-esteem. The importance of reliable support through trustful relationships in health encounters was further emphasized due to previous experiences of feeling misunderstood and criticized in life. Feeling listened to without being judged made the women comfortable discussing SRH. A reliable structure when meeting health care was also perceived to improve availability and support. Providing a clear structure in health care encounters was described to benefit concentration and the assimilation of information. The right organizational conditions, like sufficient education for HCPs about SRH and ADHD, having routines for asking the women about SRH and collaboration between clinics were further perceived to build reliable support that could promote SRH in the women.

Similar to other young adults, our results described that young women with ADHD often use the Internet to seek information and advice about SRH (Freeman et al., 2017), especially when friends lacked understanding of how the diagnosis affected relationships. However, our results revealed that even though information concerning sexuality and relationships was available online, such resources were not always able to provide answers to questions of how ADHD could influence SRH. These results indicate a need to improve evidence-based online information targeting SRH and ADHD. The importance of providing reliable Internet-based sources, exemplified in our study as podcasts, apps, and websites, was further described by Mitchell et al. (2014) revealing how information about sexual health online was valuable and more common in sexual minority groups who lacked suitable information off-line or felt like they did not have anyone to ask. Internet-based sources were also described in the study to provide anonymity, which could be especially important for young women with ADHD as they could be at risk of self-stigma and fear of being judged in clinical encounters due to sexual norm-breaking (Masuch et al., 2019; Young et al., 2020). The use of artificial intelligence may also be considered as chatbots, and virtual conversational agents may deliver information in a manner that can feel more private and non-judgmental, overcoming access barriers to stigmatized areas like sexual health (World Health Organization, 2024). However, even though the Internet can be seen as an easily accessible source of information and advice, our results emphasize that online sources only provided support to a certain extent, possibly reflecting a lack of reliable Internet sources. However, considering the high rates of STD, teenage pregnancy, and sexual victimization among young women with ADHD (Chen et al., 2018; Skoglund et al., 2019; Snyder, 2015), accessible health care clinics providing professional support may also be particularly important for these women.

To further understand and manage one’s sexuality, both women and HCPs in our study concluded that it was important to receive an ADHD diagnosis early in life. An early diagnosis could help to prevent low self-esteem and develop strategies to handle sexual situations and relationships in young adulthood. Medical treatment for ADHD was described in our result to reduce symptoms of ADHD and sexual risk-taking, which is in accordance with previous research revealing how medical treatment could prevent STDs and unplanned pregnancies in young adults (Chen et al., 2018; Hua et al., 2021). However, our findings also emphasized the importance of including partners in counseling at an early stage and educating parents about SRH and ADHD. These results were strengthened by Huggins et al. (2015), who identified how a qualitative mother–child relationship could reduce the number of sexual partners in young adults, and Wallin et al. (2022), who described that well-functioning communication with a partner could help young women with ADHD to form high-quality intimate relationships. Several HCPs in our study further suggested that discussions about sexuality could be included in already existing psychoeducational groups provided by psychiatric clinics. This could be a sufficient method to raise awareness and understanding of ADHD and sexuality at an early stage, as psychoeducational groups are empirically validated as a method to increase knowledge of living with ADHD and psychological well-being (Hirvikoski et al., 2015, 2017). The women in our study further confirmed the advantage of group discussions with people with a similar diagnosis as these could provide helpful advice and enable a better understanding of their sexuality. Offering several options for early support as suggested in our results may be important to promote SRH, considering that research favors a multimodal and multidisciplinary approach to improve the functional outcome of living with ADHD (Kooij et al., 2019).

Our results indicated that, according to both women and HCPs, a trustful relationship in clinical encounters was important to promote SRH. While trust was perceived in our findings to make the women comfortable discussing their sexual experiences and more likely to return for STD or pregnancy testing, Gaebel et al. (2014) revealed that trust influenced the chances of seeking health care, compliance with treatment, and patient satisfaction in the context of mental health services. Considering that young women with ADHD present with a higher risk for sexual victimization and relational problems (Bruner et al., 2015; Snyder, 2015), building trustful relationships with HCPs may therefore be especially important. However, building trust in health encounters was perceived to be influenced by previous experiences of feeling criticized and misunderstood in school and by health care according to our findings, possibly reflecting experiences of stigmatization or discrimination reported by adults with ADHD in several areas in life, including health care (Masuch et al., 2019). Because women with ADHD may mistrust HCPs, efforts to improve trust in the professional encounter may be particularly important. Our findings suggested routinely offering follow-up appointments and longer counseling sessions, preferably with the same HCP in health care clinics. These results were strengthened by previous studies emphasizing that trust is influenced by continuity in treatment and having time to get to know one another (Gaebel et al., 2014; Unis et al., 2021). Trust was further perceived by both women and HCPs, in our study, to be built on non-judgmental encounters, involvement in one’s care, and HCPs trying to understand the women’s whole life situation. These elements are recognized in the concept of person-centered care, where listening to the person’s narrative is the first step in establishing an equal partnership and shared decision-making is an aspect of finding personal resources and skills (Ekman et al., 2011). To endorse trustful relationships, recording the women’s preferences, beliefs, and values, along with their involvement in care and treatment decisions, may according to Ekman et al. (2011) further validate their perspectives, enhance transparency in patient–provider interactions, and promote continuity. To further promote participation in care, our results also suggested that youth clinics and gynecological clinics had to be flexible to enable the women to be involved in their own care by providing, for example, drop-in appointments, attending to women without having an appointment or sending SMS reminders. Even though accessible clinics may be important for all young adults, flexible clinics may particularly benefit adults with ADHD as they are more prone to missing appointments in health care (Soendergaard et al., 2016), possibly reflecting challenges with planning and organization associated with ADHD (American Psychiatric Association, 2013).

Our results further emphasized that women with ADHD rely on the health care system to receive support regarding SRH. To make support accessible and to help the women assimilate information, our findings revealed a need for a clear structure in both clinical encounters and on an organizational level. Even though all young adults may benefit from a clear structure in health encounters, challenges with inattention described in our findings are associated with having ADHD (American Psychiatric Association, 2013). In accordance with Klint Carlander et al. (2022), describing contraceptive counseling in young women with ADHD, adapted appointment times, reduced stimuli in the environment, and a variation of communication tools may be particularly important for them. Providing organizational conditions like enough time for appointments and education concerning ADHD and SRH may also influence how and if support is given, according to our results. Knowledge about ADHD among HCPs working in youth health and gynecology was revealed to improve understanding of the women’s sexual behaviors. Educational efforts concerning ADHD and SRH targeted at HCPs in sexual health clinics may therefore help clinics individualize care and reduce stigma associated with ADHD and norm-braking sexual behaviors in women (Young et al., 2020). Educational efforts concerning SRH may also be offered to HCPs in psychiatric clinics, considering that lack of knowledge concerning SRH and doubt about clinical skills were perceived to prevent HCPs in psychiatric clinics from raising questions concerning SRH with the women, similar to results found concerning nurses working in mental health clinics (Quinn et al., 2013). However, having clinical routines involving always asking young women with ADHD about SRH in general in psychiatric clinics was perceived to make it easier to raise the subject. It may be especially important to raise questions concerning SRH in these clinics as some women described having longer trustful relationships with HCPs in psychiatric clinics.

Overall, HCPs in our study described a need for a more comprehensive care to promote SHR as sexual behaviors and relations were not a separate part of life but integrated and influenced by the whole life situation. Collaborative initiatives between clinics were mentioned to promote understanding an accessibility to care. Even though national Swedish guidelines on how to provide care and support for individuals with ADHD, not specifically target SRH, collaboration between health care institutions, schools and social care are recommended (Swedish National Board of Health & Welfare, 2022). However, examples of collaboration models in student health care and maternity care have been seen to improve patient satisfaction with and accessibility to mental health services through coordinating care managers and psychiatric consults (Reist et al., 2022). It is also possible that implementing standardized protocols could improve communication and coordination within a clinic or between clinics providing a more efficient and accessible care for the women.

### Strengths and Limitations

The strength of this study was that it was the first study, known to us, describing conceptions of how to promote SRH in young women with ADHD among a varied sample including women with a diagnosis and HCPs from clinics dealing with youths’ and women’s health and psychiatry. The study provides insights that can contribute to knowledge on how to promote SRH in young women with ADHD. A limitation of the study may be the relatively small sample size. However, the present sample size of 31 was seen to provide a rich description of varied conceptions of how to promote SRH. According to Marton and Booth (2000), there are limited ways of conceiving a phenomenon within a group and variations will most likely be covered with a sample size of 20–25 participants. It is also necessary to consider that the result may not reflect all women with ADHD. Individual variations of ADHD symptoms, functional ability, and comorbidity need to be considered when transferring data.

### Conclusion

Having reliable support may be particularly important for promoting SRH in young women with ADHD. Having easily accessible information that targets both SRH and how ADHD can influence sexual relationships and behaviors may be important. Written and oral information concerning SRH and ADHD may be distributed by psychiatry and sexual health clinics as well as by Internet sources. Early assessment and diagnosis of ADHD could further enable early access to SRH counseling, psychoeducation, and medical support, helping to prevent sexual risk-taking behaviors and strengthening self-esteem in sexual relationships. Clinics may raise the level of education about SRH and ADHD among HCPs and provide time for appointments, as well as initiating collaboration between clinics. This can make support accessible and help build trustful relationships that are based on non-judgmental meetings and involvement in one’s care. HCPs, regardless of clinical belonging, can bear in mind that the varying components of reliable support are important. However, future studies need to explore how intervention programs promoting SRH guidance, educational efforts for HCPs, and collaborative care can be developed and implemented in clinical practice.

## Data Availability

Raw data will not be publicly available to uphold the signed confidentiality statements and privacy of participants.
